# Micronutrient inadequacy in Europe: the overlooked role of food supplements in health resilience

**DOI:** 10.3389/fnut.2025.1686365

**Published:** 2025-09-24

**Authors:** Samantha Christie, David Crooks, Rowena Thomson-Selibowitz, Ashley Green-Woolard, Konstantinos Mantantzis

**Affiliations:** ^1^Council for Responsible Nutrition, UK (CRN UK), Coventry, United Kingdom; ^2^Nestlé Health Science, Vevey, Switzerland; ^3^Amway Corporation, Ada, MI, United States; ^4^Department of Regulatory, Medical, Safety, Quality and Compliance (RMSQC), Bayer Consumer Care AG, Basel, Switzerland

**Keywords:** Europe, food supplements, health resilience, micronutrients, nutritional gaps, nutrition policy, public health, vitamin and mineral insufficiency

## Abstract

Micronutrient inadequacy is a significant issue in Europe, partly driven by an evolving food landscape. Food supplements play a critical role in offsetting these gaps, particularly in vulnerable groups, and contribute to the overall health resilience, wellbeing, and productivity across the life-course and population. However, both the extent and impact of micronutrient insufficiency and the essential role of food supplements remain underrecognized in public health, clinical training, and practice. We examine the reasons behind this widespread under-acknowledgment, along with selected evidence demonstrating the tangible benefits of food supplements in bridging micronutrient gaps and fostering health resilience. We discuss the health policy implications of incorporating food supplements into public health strategies to enhance nutritional status, help reduce the risk of chronic diseases, improve workforce productivity, and reduce healthcare costs across Europe. Ultimately, we call for an integrated approach to nutrition policy that fully recognizes and utilizes the value of food supplements in supporting a healthier and more resilient European population.

## 1 Introduction

Food supplements are concentrated sources of micronutrients, or other substances with nutritional or physiological effects, marketed in the form of tablets, capsules, powders or liquids in measured doses (1). These were developed in response to an evolving understanding of the importance of micronutrients to maintain physiological homeostasis and good health (2), as well as the growing awareness that supplementation would be needed to address gaps in dietary intake that could lead to micronutrient deficiency, with later recognition that food supplements could support resilience and optimal health (3–5). Food supplements are regulated as foods in Europe, to ensure that they can effectively bridge dietary gaps without posing health risks (1).

Despite the direct and multifaceted relationship between micronutrient status and health (6), the prevalence of low micronutrient intake in Europe (7), and the relatively long historical use of food supplements (3), acknowledgment of their importance among policy makers is still lacking—both in public health and clinical practice. Our present review focuses on the changing food landscape that has contributed to micronutrient inadequacies across Europe, and the evidence for the role of food supplements in helping bridge nutritional gaps and promoting health resilience in the general population and specific subgroups. Integrated nutrition policies must be developed and implemented to acknowledge the public health crisis caused by micronutrient inadequacy in Europe *and* the tangible positive impact of food supplements. Vitamin D will be used as a case in point, considering its critical roles in health (8), the insufficiency of European dietary supplies compared to Reference Intakes (RI) (9–12), the prevalence of suboptimal serum 25-hydroxyvitamin D (25(OH)D) levels throughout Europe (13), and the consequences of vitamin D dietary insufficiency and deficiency (14).

## 2 Balanced nutrition is essential for public health

Nutrition has long been recognized to be a cornerstone of human health—a critical foundation for growth and vitality. Balanced nutrition, which includes an adequate supply of micronutrients, underpins immune resilience and supports cardiometabolic function, reproductive health, cognitive and brain development, and overall physical and mental health (15). The need for a sufficient intake of micronutrients extends throughout life—from preconception, fetal development, and infancy (16), to early childhood and adolescence (17), and continues into adulthood and old age (18, 19). Dietary reference values were designed to safely prevent micronutrient deficiencies in different groups of healthy individuals (20); however, these levels may not be sufficient to promote *optimal* health (19, 21).

Poor nutrition has a substantial impact on society by increasing strain on healthcare systems, reducing resilience to health crises, and leading to higher rates of chronic diseases and shorter life expectancies—even in the developed world, where micronutrient-poor food is abundant (22–25). It has been suggested that the presence of one or more micronutrient deficiencies should be considered in all chronic diseases, particularly in vulnerable groups (6, 26). The World Health Organization has highlighted the economic burden of poor nutrition, estimating that addressing nutrient insufficiencies could save billions in healthcare costs globally (27, 28).

## 3 Current nutritional needs and gaps within Europe

To maintain yield and ensure food security, modern agricultural practices have needed to adapt to increasing demand, environmental pollutants, and climate change (24, 29). The subsequent depletion of micronutrients in staple crops, along with the ready availability of low-micronutrient convenience foods, has contributed to an insufficient availability of micronutrients in the food supply (30, 31).

Despite the abundance of food in high-income countries, nutrient inadequacy is a significant issue, with many individuals not meeting the recommended intakes for essential micronutrients, especially among certain demographics (7, 32–34). For example, vitamin D deficiency is well established in Europe and has been described as reaching “pandemic” levels, affecting up to 40% of the population (13). A low vitamin D status can lead to an increased risk of fatigue, muscle aches, pains and weakness, infections, osteomalacia, rickets, osteoporosis, falls and fractures, and may contribute to autoimmune disorders such as type 1 diabetes (14, 35). In addition, widespread insufficiencies have been reported across Europe for vitamins A and B12, folate, iron, calcium, iodine, magnesium, and zinc, particularly in vulnerable populations (Table 1). The decreased availability of micronutrients in the European food supply has led to an increasing reliance on food supplements to maintain micronutrient sufficiency—in 2022, over 90% of over 13,000 people questioned in 14 countries reported using a food supplement within the last 12 months (36). The true prevalence of micronutrient deficiencies in Europe, especially in high-risk populations, is one of the key initial aims of the ongoing Zero Hidden Hunger EU initiative (37, 38).

**Table 1:** Overview of various vulnerable groups at risk of selected micronutrient insufficiency and deficiency in Europe.^a^

	**Micronutrients at risk of inadequacy; selected examples**								
	**Vit A**	**Vit D**	**Folate**	**Vit B12**	**Ca**	**Mg**	**Fe**	**Zn**	**I**
**Main functions**	Vision, immune functions, skin and mucous membrane integrity, cell growth and development	Bone health, immune functions, cell growth and function, muscle function	RBC formation, fetal neural tube development, DNA synthesis and repair, cell growth and function, amino acid metabolism	RBC formation, nerve function, DNA synthesis, energy production, cell growth and function, immune function	Bone and tooth health, muscle contraction and relaxation, nerve conduction, cellular metabolism, blood clotting (wound healing)	Muscle and nerve function, electrolyte balance, bone health, protein and DNA synthesis, energy production, cell growth	Oxygen transport, RBC production, energy production, cellular function	Immune functions, cell protection, acid/ base metabolism, growth and development, DNA and protein synthesis, cognitive function, fertility and reproduction, vision	Thyroid hormone production, metabolism regulation, growth and development, nerve function
**Groups**									
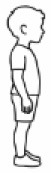 **Children** (9, 67, 84)	↓	↓	↓	—	↓	—	↓	↓	↓
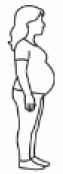 **Pregnancy and lactation** (85)	↓ (lactation only)	↓ (pregnancy and lactation)	↓ (pregnancy and lactation)	↓ (lactation only)	↓ (pregnancy and lactation)	↓ (pregnancy and lactation)	↓ (pregnancy)	↓ (lactation only)	↓ (pregnancy)
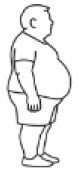 **Obesity** (86)	↓ (incl. after bariatric surgery)	↓ (incl. after bariatric surgery)	↓ (incl. after bariatric surgery)	↓ (incl. after bariatric surgery)	↓ (incl. after bariatric surgery)	↓	↓ (incl. after bariatric surgery)	↓ (only after bariatric surgery)	—
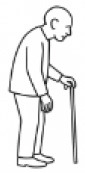 **Older adults** (84, 87, 88)	↓	↓	↓	↓	↓	↓	↓	↓	—
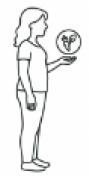 **Vegetarians and vegans** (89, 90)	—	↓	↓ (older adults, pregnancy and lactation)	↓	↓	—	↓ (children, adolescents, pregnancy and lactation)	↓	↓

^a^Evidence of multiple micronutrient dietary insufficiency has also been demonstrated in mid-life adults in the UK (20–50 yrs) (91).

Key: ↓, deficient/insufficient; Ca, calcium; Fe, iron; incl., including; I, iodine; Mg, magnesium; RBC, red blood cells; Zn, zinc; Vit, vitamin.

## 4 Lack of recognition of micronutrient insufficiencies

The prevalence of dietary insufficiency and suboptimal micronutrient status across Europe, as well as the health problems caused by micronutrient insufficiencies, are under-recognized issues. Clinical training for primary healthcare providers is generally limited with respect to micronutrients and the needs of distinct cohorts, with only basic information on their role and a focus on treatment of key deficiencies such as iron and vitamin D—i.e., frank deficiency diseases (e.g., nutritional rickets, anemia, osteoporosis, osteomalacia). The long-term impact of micronutrient inadequacy on overall health resilience is an educational gap that needs to be addressed. This lack of knowledge means that healthcare providers may not see a need to routinely assess micronutrient deficiencies or consider them to be a factor in poor health. A greater emphasis on nutrition training for health professionals, including information on how to integrate micronutrient knowledge into preventive or therapeutic care, is essential to help improve population health and wellbeing (39–41).

Furthermore, nutrition research and policies for food strategies in public health often focus on macronutrient-level issues such as calorie intake and risk of obesity (42). The role of micronutrients is often overlooked, apart from those with clear, large-scale evidence, such as vitamin D for rickets or folic acid fortification to reduce the risk of neural tube defects during pregnancy (43). This lack of acknowledgment of the significant impact of multiple micronutrient insufficiencies on public health has led to insufficient funding and research in this area. It is only recently, for example, that awareness has begun to change with respect to the target RI; these were primarily designed to prevent deficiency—but may be too low for optimal health across different populations (19, 21). For example, in the case of vitamin D, it has been suggested that the RI is insufficient to fully support immune function, muscle function, fracture risk, cardiometabolic health, and those with underlying health conditions that predispose them to vitamin D deficiency, including obesity (44, 45).

## 5 Food supplements are vital to bridge nutritional gaps

Eating a healthy, varied diet is the foundation to good nutrition and a fundamental self-care measure for good health (46). Yet in practice, it is not always possible to consume an adequate quantity of the right foods every day to satisfy micronutrient needs (32). Furthermore, simply preventing micronutrient deficiency is not always sufficient to provide health resilience and optimal health or reduce the risk of chronic disease for all (19, 21). Additionally, it may not be feasible to achieve sufficiently high micronutrient intakes required via diet alone, particularly in those at risk of micronutrient insufficiency such as children, teenagers, pregnant and breastfeeding women, the elderly, and those with restrictive diets (Table 1) (32). Therefore, the use of a food supplement alongside the daily diet can help to address nutritional gaps and mitigate the problems caused by suboptimal nutrition.

### 5.1 Impact on individuals

As the global life expectancy continues to increase (47), it is vital that individuals take steps throughout life to remain healthy into older age to minimize the risk of age-related chronic non-communicable diseases such as obesity, type 2 diabetes, and hypertension (48). Nutrition has an impact on every aspect of health, and micronutrients are essential for homeostasis and physical health, cognitive function, wellbeing, and stress resilience (15). Food supplements can play a key role in self-care to support and maintain overall health, with the potential to help reduce the risk of chronic diseases.

For example, vitamin D supplementation (especially in autumn, winter, and early spring, i.e., before the beginning of April) is recommended to avoid the occurrence of rickets and osteomalacia in northern and western Europe (49). Daily use of vitamin D supplements (50 μg; 2,000 IU) throughout the year is recommended by some for the general adult population to achieve and maintain adequate vitamin D status and prevent deficiency (50). It has been estimated that supplementation with vitamin D plus calcium in adults with osteoporosis would prevent more than half a million fractures each year in the European Union (EU) alone (51). In addition, research is showing that enhanced vitamin D status might help to reduce the risk of upper respiratory tract infections (52), and optimizing vitamin D status may reduce the risk of type 2 diabetes and improve outcomes for those with chronic health conditions (53, 54). As such, food supplements can play a critical role in promoting health across different populations. Depending on the desired outcome, different levels of supplementation may be required—highlighting the need for flexibility in supplementation regimens and the importance of supplements as a tool for achieving and maintaining resilience and optimal health.

### 5.2 Impact on healthcare systems and economies

In addition to the individual health benefits that could be achieved by bridging micronutrient gaps with food supplements, there could be a considerable socioeconomic impact in the form of reduced healthcare costs, greater productivity, and associated long-term savings.

For example, vitamin D facilitates the intestinal absorption of calcium and phosphorus (both required to form the bone component, hydroxyapatite) and interacts with parathyroid hormone to stimulate the renal reabsorption of calcium and to activate osteoclasts (responsible for bone resorption); vitamin D deficiency leads to impaired bone mineralization (55). Using vitamin D plus calcium supplements to help to address fractures in adults with osteoporosis in the EU could save approximately €5.7 billion every year; i.e., €5.58 saved for every €1 spent on calcium and vitamin D (51). Vitamin D supplementation may also be cost-effective in avoiding rickets in children and osteomalacia in adults (56), as well as type 2 diabetes in adults (57). In fact, it has been estimated that raising the mean concentration of 25(OH)D at the population level could reduce the incidence and mortality rates for eight of the top ten leading causes of death in the US, as well as improve adverse pregnancy and birth outcomes (44). Furthermore, better nutrition leads to a healthier population that is more productive and less prone to absenteeism (58, 59), with a consequent boost in economic growth (60). Supplementation with vitamin D, for example, could enhance productivity in the workforce (61). Over the long term, cost-effective national and regional preventive strategies such as food supplements could help to reduce the risk of chronic disease, improve health-related quality of life of individuals, relieve the nutrition-related financial burden on healthcare systems, and subsequently contribute to European economic growth.

## 6 Future perspectives

Research into the scale of the true prevalence of micronutrient insufficiencies across Europe will be a first step to address the lack of widespread awareness of the problem. The Zero Hidden Hunger EU initiative aims to provide better data on the prevalence, causes, and costs of micronutrient deficiencies across different regions and populations—including strategies to communicate this information to policymakers (62). This will help to inform public policy to eradicate micronutrient deficiency within Europe (37). It is also important to increase recognition that the evolving nature of the food landscape has a big impact on the prevalence of micronutrient deficiencies (24, 30). The adverse effects of climate change on nutrient levels in crops (63), for example, as well as changes in dietary consumption patterns from an omnivore diet to one that contains more plant-based food (64) and the reliance on convenience foods (65), may increase reliance on food supplements to maintain micronutrient sufficiency.

There is a need to implement proactive nutritional strategies that will promote optimal health rather than simply prevent micronutrient deficiencies (19, 21, 66). To achieve this, it will be necessary to devise appropriate dietary recommendations that better support overall health and wellbeing. The EU's “Food 2030” strategy (29) emphasizes personalized nutrition and the role of supplements in achieving optimal health. Future research should acknowledge that people require different levels and combinations of nutrients to adequately support their own optimal health—a complex issue that could be dependent on factors such as genetics, lifestyle, nutrient interactions, and other interdependent factors (67–69).

In the meantime, nutritional policies should include fortifying foods with essential micronutrients [such as folic acid (70), vitamin D (71, 72) or iodine (73–76)] and encouraging the safe use of food supplements (1, 77) to enhance nutritional status and thereby help to reduce the risk of chronic disease and minimize healthcare costs. These actions are necessary complements to dietary improvement efforts and will help to ensure a healthier, more resilient population across Europe.

Identifying those specific individuals who would benefit most from a food supplement is also essential. In the case of vitamin D, guidelines specifically target infants and young children, pregnant and breastfeeding women, people over 65, people who have low or no exposure to the sun, and people with dark skin (78). However, it is likely that most individuals would benefit from daily supplementation, particularly during the autumn, winter, and spring months—including those with conditions that predispose them to vitamin D inadequacy (50). Thus, an integrated approach for food supplements in nutrition policy is required, to ensure that the right individuals benefit from micronutrient supplementation.

To increase awareness that food supplements can help to bridge micronutrient gaps, it is vital to include more comprehensive information about the prevalence, impact, and management of micronutrient insufficiencies in the training of healthcare professionals, including how to implement this knowledge and the importance of nutritional self-care for the general public (4, 39).

### 6.1 Challenges in achieving optimal health and nutrition

Defining optimal health is challenging. It is often described as being a state of complete physical, mental, and social wellbeing, not merely the absence of disease—a broad definition that lacks specificity. Factors such as demographics, genetic predisposition, environmental and lifestyle factors, and pre-existing conditions vary between individuals, making it difficult to establish concrete guidelines for achieving optimal health. These factors also influence specific micronutrient requirements, further complicating the establishment of universal dietary recommendations for optimal health. Ideally, adaptive frameworks would need to be developed to enable personalized nutrition that can accommodate individual differences without oversimplifying health outcomes—a task that requires much more extensive research.

Current models for determining micronutrient needs often rely on population averages, which may not accurately reflect the needs of specific individuals. These models can overlook the nuanced interactions between micronutrients and individual health conditions. Widespread micronutrient assessment in the general population would confirm specific micronutrient needs, but any measures used would need to be accurate, standardized, quick to perform, cost-effective, and provide rapid feedback to facilitate their acceptance and adoption in clinical and public health settings (19).

Another challenge is the methodological aspects employed in some lower-quality clinical micronutrient intervention studies. For example, inadequate trial design can mean that results are less than useful for advancing our understanding of the relationship between micronutrients and health outcomes (44). It is well-established that vitamin D is crucial for bone health (79), and some intervention studies have indicated that it has beneficial effects on bone (44). Yet negative results from other studies can lead to a lack of confidence when the totality of the evidence is assessed (80). A common methodological oversight may explain the conflicting results. In this instance, it is now understood that the nature of intervention—i.e., high-dose bolus vitamin D (e.g., 100,000 IU in one dose), as opposed to a more physiologically-favorable daily lower dose of vitamin D—can lead to false negative outcomes or nil treatment effects. The current consensus is that pharmacological-strength bolus doses of vitamin D may be limited in their applications in these type of research settings (81, 82).

In addition, researchers conducting meta-analyses require access to the appropriate datasets to scientifically determine the effects of micronutrient supplementation. Analyses that use individual participant data are a more favorable form of data to capture the extent of the between-individual variability in outcomes (83). Lastly, strategic data sharing and generation will also help to improve the quality of research into both individual and public health benefits of food supplements.

## 7 Conclusions

Good nutrition is essential for health, resilience, and wellbeing. Food supplements are critical in bridging the gaps between insufficient dietary intake and requirements among populations and individuals. They can play an important role in enhancing individual and public health while supporting productivity and economic growth across the life-course. The under-recognition of micronutrient insufficiency among policymakers, public health officials, and healthcare providers hinders efforts to fully address this issue through easily available, cost-effective measures such as food supplements.

To effectively promote optimal health, it is crucial to recognize that nutritional needs cannot be met using a one-size-fits-all approach. As we face geoclimatic changes, evolving food systems, and varying micronutrient supplies in our diets, it is important to ensure that food supplements are adaptable to meet individual requirements, based on demographics, health conditions, and lifestyle choices. The need for flexible supplementation is underscored by the fact that micronutrient deficiencies and insufficiencies can vary widely across regions and populations. Policymakers and health professionals must focus on adaptive strategies that prioritize personalization in nutritional guidelines. This approach will involve ongoing research to better understand the interplay between genetics, lifestyle, and nutrition, enabling the development of tailored supplementation options that can effectively support diverse populations to achieve optimal health.

By incorporating food supplements into public health strategies, we can ensure better nutritional outcomes and reduce the risk of chronic diseases and strain on healthcare systems. A more comprehensive and integrated approach to nutrition policy and clinical training is needed, where food supplements are not seen as a replacement for dietary improvement, but as a necessary complement to foster healthier and a more resilient population and economy in Europe.
